# Hospital Reports—Hospital Elections: Sir. A. Carlisle's Letter—Dissentions in the Surgical Cabinet

**Published:** 1829-04-01

**Authors:** 


### XLII.

### Hospital Reports—Hospital Elections.

Sir Anthony Carlisle, President of the College of Surgeons, has published the following letter in the Times newspaper, which he considers the best medium for public appeals. It may be so for one part of the address—that which is directed to the Governors of Hospitals; but that part which appeals to surgeons, and relates to the publication of hospital reports, would be better conveyed through the regular professional channels. Our readers are aware that we are no very great admirers of Sir Anthony Carlisle's lucubrations and publications in general. But we confess that there appears to be much less of eccentricity, and much more of rational and practical utility in this letter than in most of those which have preceded it. But before proceeding further, we beg our readers to carefully peruse the document itself.

TO THE EDITOR OF THE TIMES.

Sir,—As your newspaper may be justly considered to be the very best medium, for public appeals, I solicit the insertion of the following hints to the Governors of Medical Hospitals. Those institutions having become schools for professional instruction, the rich as well as the poor have an unceasing interest in all their ministrations. After thirty-six years of experience as an hospital-surgeon in the metropolis, I may perhaps be allowed to state my thoughts upon the better regulation of their surgical department. The election of persons destined to become public surgeons is at all times an important and difficult task, for the electors are generally perplexed between the applications of personal friends and professional eulogists.

If the office of an hospital surgeon were a mere money-making affair, the choice of the electors would be of no public interest; but it is otherwise, for those elections confer public character, and as they are well or ill decided, so will be their influence upon the healing art. Under these impressions, I submit the following proposals for the better regulation of the surgical appointments in hospitals, viz.

That there should be three classes of surgeons in every general medical hospital. The junior class to be styled assistant surgeons, who shall not be eligible for that office until the completion of twenty-five years of age, nor if they have any defect in the eyes, or be obliged to use glasses. The second class to be styled principal surgeons, each of whom should be thirty years of age at the least, having been previously an assistant-surgeon in the same hospital for three years, and to remain principal or. operating surgeons until they respectively arrive at the age of sixty years; afterwards they shall be eligible to become consulting surgeons, and cease to do those operations which demand the utmost precision.

That there shall be one principal surgeon and one assistant surgeon allotted to take the charge of every fifty promiscuous in-door patients which the hospital may be capable of receiving.

The especial duties of assistant-surgeons to be, the daily care of the in-patients under the direction of their respective principal surgeons, the attendance upon accidents and out-patients, and the preparatory arrangements for all dangerous operations.

In every case of proposed dangerous operation, or of one of questionable expediency, the principal surgeons and the consulting surgeons to hold a conference in the presence of their pupils, and determine by a majority of the votes of such surgeons the measures to be adopted.

If the foregoing suggestions were made the standing orders for medical hospitals, every grade of society would feel assured of the competency and of the relative fitness of public surgeons; the profession would be freed from many desperate adventurers, and the art of surgery would assume the dignity of a noble profession devoted to the deepest interests of humanity.

To prove that this exposition is now demanded, I subjoin an address to the hospital-surgeons of England, and which I have not been able to carry into effect, although I have laboured against its sluggish opponents for eight years. I am therefore at last compelled to submit the measure to public opinion, under a hope that some powerful influence may command its adoption. Many of your readers will be startled to learn that the most violent objector to those hospital reports, is the child and champion of surgical demagogues. He argues "that such reports would be of no use;" that "the hospital surgeons of England hate their own college too ardently to expect any co-operation from them;" that "the style of the address would be disgraceful to the College;" and lastly, "that such reports would only display the comparative mortality in the different hospitals," —a fact which I consider to be of the utmost importance to the public, the best security against unjustifiable surgical operations, and the highest incentive to honourable competition. I am, Sir, your obliged servant, ANTHONY CARLISLE.

*Langham-place, Jan.* 20.

PROPOSED ADDRESS TO SURGEONS OF EVERY GENERAL MEDICAL HOSPITAL IN LONDON AND IN THE KINGDOM OF ENGLAND.

"The President, the Vice-Presidents, and the members of the Council of the Royal College of Surgeons in London, have the honour to solicit your co-operation in a public measure, which promises many professional advantages.

"The College have often announced a desire to collect documents for a series of transactions, where the progressing improvements in surgery, and in its auxiliary sciences, may be suitably published.

"One of the principal impediments to the interchange and to the diffusion of surgical knowledge, appears to have been the want of liberal and frequent communication among hospital practitioners, who may be justly presumed to possess the best opportunities, and the most extensive experience.

"By adding the valuable results of your public practice to the contributions of the other English hospitals, the College expect to obtain a regular continuation of authentic and impartial evidence, which may do honour to our profession and improve every branch of the healing art.

"With a view to spare trouble, and to give uniformity to the intended reports, we enclose the form of a tabulated register, to which should be affixed the signatures of all the principal surgeons as a verification of its contents.

"In this appeal to your public liberality, the College rely on your cordial compliance, since the proposed summary of hospital practice will not interfere with individual authorship; while it must present to the world a generous union of our efforts to promote and to improve our profession.

"A printed copy of each half-yearly report will be sent to the surgical library of every contributing hospital.

"The College will be obliged by receiving your answer before the 1st of January ensuing.

"Royal College of Surgeons in London, day of 18 .

"SKETCH FOR TABULATED RECORDS.

| Initials of Names. | Age and Sex. | Apparent Constitution. | Disease or Injury. | Treatment. | Result. |
|---|---|---|---|---|---|
| | | | | | |

"In addition to the providing and keeping tabulated accounts of the principal cases under your care, it is desirable that you should annex every peculiar occurrence, vicissitude, or remarkable accident, whether favourable or otherwise, not reducible to the above tabulated form; and also mention the general methods of particular treatment which seem to denote the juvantia or ledentia in your practice; and, as each of the several public hospitals of this kingdom possess particular formulæ, or employ certain medicines in doses or forms imperfectly known to the profession, the College will be glad to receive all such information, whether sanctioned by individual authority or by general experience."

The division of the Chirurgical Hospital Corps into three bodies, assistants, operatives, and consultants, may appear too complicated or refined to some of the surgical profession—and, for reasons not very difficult of appreciation, the plan will meet with much opposition among the existing race of hospital surgeons. The sexagenarian thinks his eyes as good, and his hand as steady, as when he was only half that age—and therefore *he* will not consent to lay aside the knife, just when he considers himself as in prime condition for wielding it with profit to himself and advantage to the patient. In respect to the operatives, there is little doubt of a general opposition among them against the introduction of assistants, who are to tread upon their heels, and share the honours as well as the emoluments of office. Yet we verily believe that the regulation proposed by Sir A. Carlisle would be beneficial to the public and to the profession at large. We have now the authority of Sir Astley Cooper for the propriety, nay necessity, of assistant surgeons to public hospitals; and we hope the example of Guy's will be followed by every hospital in England.

To the proposal for collecting and publishing hospital reports, we see no rational grounds for objection. We cannot indeed approve of the manner in which Sir Anthony has alluded to Mr. Lawrence in the above letter. Mr. Lawrence is one of the council of the College, and whatever may have been his opinions respecting surgical REFORM a few years ago, it is abundantly evident that he has now no connexion with certain surgical REFORMERS who shall be nameless on the present occasion. We do not therefore deem it quite fair or generous to designate Mr. L. as the "child and champion of surgical demagogues." We believe Mr. Lawrence is perfectly sick and ashamed of the veritable—"child and champion of demagogues," else he would never have entered the councils of the College. But we do think that Mr. Lawrence owes it to the profession to answer the allegation made against him in Sir A.'s letter. We can scarcely believe it possible that Mr. Lawrence, of all men, should object to the attempt of the College to excite the public functionaries of hospitals to a publication of their experience. Unless they are excited to this attempt by some Public Body, no attempt will ever be made—and what Body is more proper for the concentration and publication of these reports than the College of Surgeons? We hope and trust that the College of Physicians also will set something of this kind on foot, for the promotion of science, and the general respectability of the profession.

We cannot close this notice of Sir Anthony's letter, without deploring the evidently distracted state of the Council—and, consequently, of the deliberations of the Royal College of Surgeons! When the President of the surgical cabinet finds, or thinks it necessary to appeal to the public through the medium of a newspaper, and to convey harsh expressions of censure on one of the Council, it may well be inferred, that we have a "divided cabinet" in Lincoln's-Inn-Fields, as we used to have in Windsor Castle! We want a WELLINGTON there! A little *military* discipline would be useful in the Temple of Chiron!

